# Retraction Note: The microRNA-1246 promotes metastasis in non-small cell lung cancer by targeting cytoplasmic polyadenylation element-binding protein

**DOI:** 10.1186/s13000-017-0637-0

**Published:** 2017-07-18

**Authors:** Weihua Huang, Huifen Li, Rongcheng Luo

**Affiliations:** 1 TCM-Integrated Hospital, Southern Medical University, Cancer Center, NO.13 Shiliugang Road, Haizhu District, Guangzhou, Guangdong 510315 China; 2 Department of Chemotherapy, Zhongshan People’s Hospital, Zhongshan, Guangdong 528400 China

## Retraction

This article [1] has been retracted by the Editor. Figure 1 (panels: A, C, D, E, F), 2 (panels: A, E, F, G, H) and 4, as well as parts of the text, were duplicated from Tian et al., 2012 [2]. The findings of this study are therefore unreliable. The Editor has been unable to confirm with Southern Medical University whether an institutional investigation has taken place. We have not been able to contact the authors.
